# Collateral Formation After Repeated Transient Dearterialization of the Rat Liver

**DOI:** 10.1155/1992/12160

**Published:** 1992

**Authors:** Li-Qing Wang, Bo G. Persson, Stig Bengmark

**Affiliations:** Department of Surgery Lund University Lund S-221 85 Sweden

## Abstract

Hepatic artery ligation is used for the palliation of patients with malignant liver tumours. Collaterals are developed rapidly and could to some extent explain why the growth is affected for only a short period. With intermittent dearterialization, collaterals seem to be avoided and possibly a more extended effect should be expected. The most efficient period of dearterialization to avoid collaterals was studied in this experiment. Five groups of rats were treated with daily repeated transient dearterializations for 0 (n = 3), 60 (n = 6), 120 (n = 6), 180 (n = 6) and 240 minutes (n = 6) respectively for 5 days and compared to another group (n = 3) that was permanently dearterialized. After treatment, celiac angiograms were obtained. All hepatic arteries were reliably occluded and patent after 5 days of daily blockades in all but two rats. There were no collaterals demonstrable on the angiograms in the first four groups after 5 days of intermittent obstruction of the arterial blood flow to the liver. After 240 minutes of dearterialization as well as after collaterals developed and were clearly demonstrated on the angiograms after six days. Liver enzymes were normal even after 4 hours of dearterialization. Repeated occlusions of the hepatic artery was reliably achieved with the implantable minioccluder. Repeated, transient dearterializations for 1, 2 or 3 hours could be performed without development of collaterals and without damage to the liver.


*HPB Surgery*, 1992, Vol. 6, pp. 105–113
Reprints available directly from the publisher
Photocopying permitted by license only

© Harwood Academic Publishers GmbH
Printed in the United States of America

### COLLATERAL FORMATION AFTER REPEATED TRANSIENT DEARTERIALIZATION OF THE RAT LIVER

LI-QING WANG, BO G. PERSSON* and STIG BENGMARK
*Department of Surgery, Lund University, Lund, Sweden*

*(Received 6 March 1992)*

Hepatic artery ligation is used for the palliation of patients with malignant liver tumours. Collaterals are developed rapidly and could to some extent explain why the growth is affected for only a short period. With intermittent dearterialization, collaterals seem to be avoided and possibly a more extended effect should be expected. The most efficient period of dearterialization to avoid collaterals was studied in this experiment. Five groups of rats were treated with daily repeated transient dearterializations for 0 (n = 3), 60 (n = 6), 120 (n = 6), 180 (n = 6) and 240 minutes (n = 6) respectively for 5 days and compared to another group (n = 3) that was permanently dearterialized. After treatment, celiac angiograms were obtained. All hepatic arteries were reliably occluded and patent after 5 days of daily blockades in all but two rats. There were no collaterals demonstrable on the angiograms in the first four groups after 5 days of intermittent obstruction of the arterial blood flow to the liver. After 240 minutes of dearterialization as well as after collaterals developed and were clearly demonstrated on the angiograms after six days. Liver enzymes were normal even after 4 hours of dearterialization. Repeated occlusions of the hepatic artery was reliably achieved with the implantable minioccluder. Repeated, transient dearterializations for 1, 2 or 3 hours could be performed without development of collaterals and without damage to the liver.

KEY WORDS: Minioccluder, repeated transient dearterialization, collateral

### INTRODUCTION

Hepatic artery ligation or dearterialization has been used for palliative treatment of patients with liver tumours since the sixties[1–3]. However, collaterals are rapidly developed and the effect on the tumour is only short lasting. They may appear within 4 days[4] and have been observed as early as 4 hours after ligation of the proper hepatic artery[5]. Using an implantable occluder, daily transient dearterializations was made possible for an extended period of time and shown to prevent the formation of collaterals[6]. Its use in patients with liver tumours has revealed some beneficial effects[7,8]. The rationale behind repeated intermittent dearterializations is to reduce the arterial blood supply to liver tumour without giving rise to a collateral circulation and to decrease the damage to the normal liver.

The swiftness with which collaterals will develop depends on the duration of the arterial obstruction. The extent and timing of collateral formation after different periods of intermittent blockade of the hepatic arterial circulation have not been

Address correspondence to: Bo G. Persson MD, Department of Surgery, Lund University, S-221 85 Lund, Sweden studied in a systematic way. We, therefore, wanted to investigate for how long the liver of a rat could be dearterialized repeatedly before collaterals would start to develop and also to measure the extent of liver damage after intermittent arterial ischemia.

### MATERIAL AND METHODS

The minioccluder (Figure 1) was made of a strip of silicone rubber sheeting (0.007 inch thick. Dow Corning, Michigan, USA) and a microballoon (Nolato, Sweden) connected to silicone tubing (0.012 ID × 0.025 OD inch. Dow Corning, Michigan, USA). The two free ends of the silicone rubber sheeting with the microballoon centered on it could be sewn together around the hepatic artery to form a ring with the required diameter, inside which the hepatic artery was occluded for a selected period. The silicone tubing from the microballoon was led out subcutaneously and fixed behind the neck of the rats where it was easily accessed. Dearterialization was achieved by the injection of about 0.08–0.1 ml saline and clamping the free end of the tubing for the desired time interval.

Thirty Sprague-Dawley rats of either sex weighing 200–300 grams were used and housed three per cage and fed standard laboratory food pellets and water ad libitum. Under ether anaesthesia the abdomen was explored through a midline incision, the liver attachments as well as the lesser omentum were dissected and all potential affluent collaterals to the liver were carefully ligated including the gastroduodenal vein, the esophageal branch and the right gastric artery. The proper hepatic artery was freed from the portal vein. The miniocclusder was placed around the hepatic artery and the silicone tubing led out as mentioned above.

The animals were then randomly divided into four groups and the hepatic artery was blocked for A: (n = 6) 1 hour; B: (n = 6) 2 hours; C: (n = 6) 3 hours; D: (n = 6) 4 hours, respectively for 5 days. In addition, one group (E; n = 3) was permanently dearterialized and one group (F; n = 3) was sham operated as control. On the sixth day celiac angiograms were obtained in all animals. The celiac artery was selectively catheterized through a femoral artery cut down. A volume of 0.6 to 0.8 ml of contrast (Isopaque, Cerebral 280 mg I/ml, Nycomed, Stockholm, Sweden) was injected by hand and the films (X-OMAT GR Film 10 × 10 cm, Kodak, Kodak-Pathe, France) were exposed at the rate of 1 film per second for 6 seconds. Liver function tests (ASAT and ALAT) were obtained from another 18 SD rats fasted overnight and subjected to dearterialization for 0 hours (n = 6), 3 hours (n = 6) and 4 hours (n = 6). Blood samples were taken at 1, 2, 8 and 24 hours after reperfusion in each group.

### RESULTS

All the rats tolerated the occlusions well which were performed in awake animals. Although they appeared disturbed at first when the balloon was inflated or deflated, they moved around in the cage and ate and drank normally. All the occluders worked well and the hepatic artery was reliably occluded immediately the balloon was inflated (Table 1). All hepatic arteries were patent in the first four groups (Figure 2) and blood flow restored immediately after deflation except in two

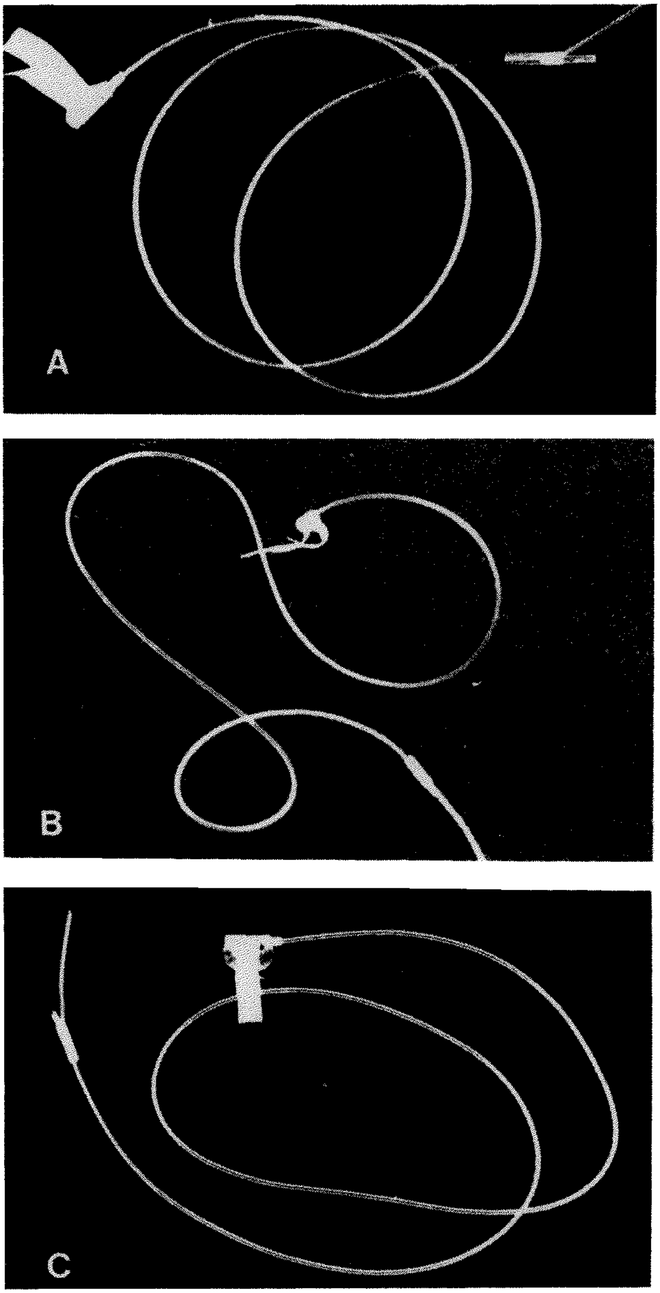

**Figure 1** A. The minioccluder is made of a strip of silicone rubber sheeting and a microballoon connected to a silicone tubing; The two free sleeves of silicone rubber sheeting have been sewn together to form a ring with the desired diameter (B) and the microballoon has been inflated with saline (C).

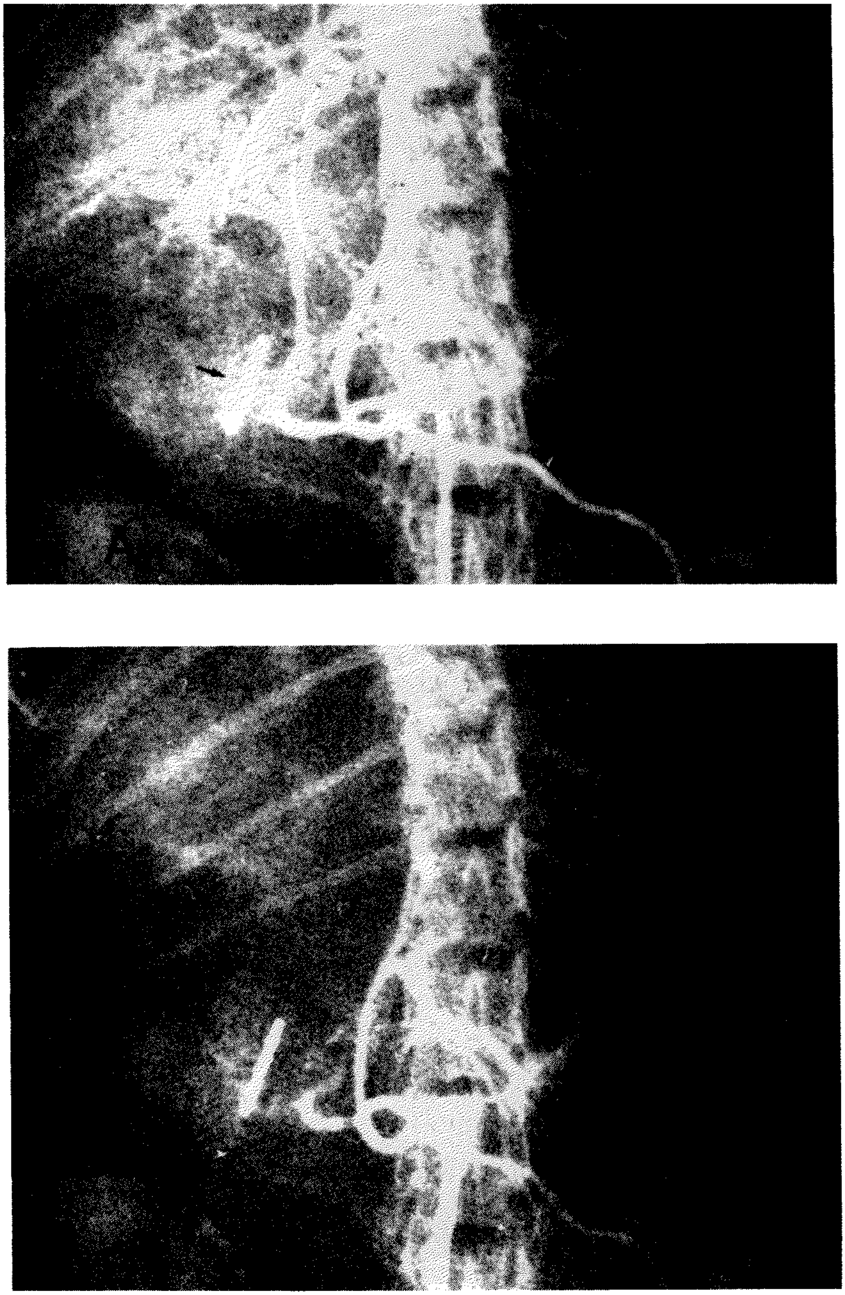

**Figure 2** Angiograms were obtained 5 days after 2 hours of repeated daily dearterializations. A: The hepatic artery is still patent and the blood flow restores immediately after deflation of the balloon. B: Effective blockade of the hepatic artery after the microballoon has been inflated with saline.

**Table 1** Angiographic results 5 days after different periods of dearterialization

| | *Transient dearterialization (hour)* | | | | | *Permanent* |
|---|---|---|---|---|---|---|
| | *0* | *1* | *2* | *3* | *4* | *dearterialization* |
| No of rats | 3 | 6 | 6 | 6 | 6 | 3 |
| Patent hepatic artery | 3 | 6 | 6 | 4 | 6 | 0 |
| Obliteration | 0 | 0 | 0 | 2 | 0 | 0 |
| Collaterals | 0 | 0 | 0 | 0 | 6 | 3 |

rats in which infection around the occluder was found. Collaterals could not be demonstrated on the angiograms in the first three groups (A, B and C). In other words, dearterializations up to 3 hours per day prevented the development of collaterals. In two rats infection around the hepatic artery led to obliteration during the course of treatment but this was not followed by the formation of a collateral circulation. Logically, arterial collaterals should eventually develop after an obliteration resembling a permanent dearterialization in this respect. However, the obliteration probably occurred late in the course of the treatment giving no time for collaterals to form before sacrifice. In contrast, collaterals were clearly seen on all the angiograms in group D and E (Figure 3). Liver enzymes after 3 and 4 hours of arterial transient ischemia were normal compared to the control group (Table 2).

### DISCUSSION

This study has shown that repeated occlusions of the hepatic artery in rats allows dearterialization without the development of collaterals. With this new minioccluder graded and intermittent reproducible blockade of the hepatic artery was possible. Daily dearterializations for up to 3 hours during 5 days did not produce any collaterals as measured by angiography. After 4 hours of daily dearterialization collaterals were developed as after permanent blockade of the arterial circulation to the liver. All arteries were patent and opened up immediately after deflation of the occluder except in 2 rats. Liver damage was insignificant even after 4 hours repeated blockades of the hepatic arterial circulation.

Development of collaterals is one of the important reasons responsible for the limited benefit of hepatic artery ligation or dearterialization to treat malignant liver tumours. A tumour may be temporarily arrested in a few days but then the growth is resumed and even accelerated partly due to a rapid formation of collaterals[4,5]. Michels defined 26 preexisting arterial collaterals through which the liver may receive blood after simple hepatic artery ligation[13]. Some of these 26 channels may rapidly establish a new flow to the liver and have been demonstrated as soon as 4 hours after ligation of the hepatic artery. The unpredictable nature of these preexistent collaterals may explain the variable effects in patients with liver tumours after simple ligation of the hepatic artery. Even after complete dearterialization, where all these potential collaterals are divided, arterial collaterals have been observed within a few weeks[14]. Hence collaterals seem to develop in response to ischemia. A crucial question is whether they can be prevented or at least reduced. Transient dearterialization has been introduced for preventing collaterals and various devices have been designed for the purpose of intermittently occluding

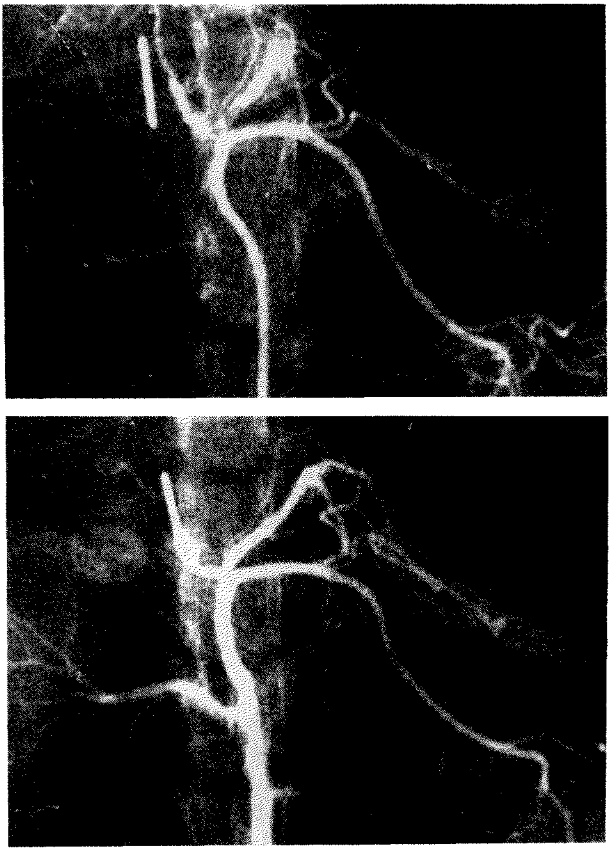

**Figure 3** A: A patent hepatic artery after 4 hours of daily obstructions for 5 days. Arrow shows the patent hepatic artery; B: Collaterals have developed and can be clearly seen (arrows).

**Table 2** ASAT (aspartate-aminotransferase) and ALAT (alanine-aminotransferase) values (mean ± SEM) pre and post different periods of transient dearterialization.

| | *Transient dearterialization* | | | | | |
|---|---|---|---|---|---|---|
| | *pre (0) post* | | *pre (3) post* | | *pre (4) post* | |
| ASAT (μkat/L) | 2.8 ± 0.08 | 2.7 ± 0.10 | 2.2 ± 0.32 | 2.5 ± 0.08 | 3.5 ± 0.18 | 3.7 ± 0.22 |
| ALAT (μkat/L) | 1.2 ± 0.06 | 1.1 ± 0.07 | 0.7 ± 0.02 | 1.0 ± 0.05 | 0.8 ± 0.08 | 0.9 ± 0.06 |

the hepatic artery. Strangulating slings placed around the hepatic artery may damage the vessel wall, resulting in thrombosis, aneurysms and other complications[9,10] and in patients dearterializations can only be performed twice[9]. In rats the animal has to be restrained in a metabolic cage with the tail fixed[10] and in this model thrombosis was frequently found after repeated strangulations (between 40 and 60% after 1 hour repeated occlusions for 5 days), and finally led to obliteration of the hepatic artery, therefore, the method seems unreliable[10]. Intraluminal balloon interruption requires heparin injections during the occlusion to prevent the early development of thrombosis of the hepatic artery[11]. Heparin injection may provoke life-threatening bleeding in patients with liver tumours who already have coagulation disorders. In addition, the pressure exerted by the balloon on the vessel wall may produce an aneurysm. In any event, leaving a comparatively big catheter inside the hepatic artery for an extended time may cause thrombosis and infection even when deflated[12]. An implantable occluder with an external occluding cuff around the hepatic artery seems to meet the requirements of such a device. The minioccluder was easy to manipulate and the animals did not have to be restrained. In contrast, they were allowed to move freely and to eat and drink during the dearterialization. Its smallness and reliability makes it useful for studies on repeated dearterializations in rats. Up to 4 hours of daily compressions of the hepatic artery for 5 days caused neither thrombosis nor obliteration which should mean that there was no significant damage to the hepatic artery. The obliteration that occurred in 2 rats was due rather to infection around the occluder than to the repeated compressions of the vessel, because all the other arteries were patent. This further confirms the superiority of this type of occluder[6–8] with a low rate of infection (6%) and thrombosis leading to obliteration if the period of occlusion is less than 4 hours. On the other hand, with an extended occlusion time thrombosis will probably develop but so will the arterial collaterals which makes this period of dearterialization unrealistic[6]. Our results showed that collaterals were avoidable when the dearterializations were performed as repeated and transient blockade of the hepatic arterial circulation for 3 hours daily. When obstruction was extended to 4 hours collaterals were seen as after permanent dearterialization. This is in agreement with our results in pigs. Thus, selecting a dearterialization period between 2 and 3 hours but performed repeatedly, should be enough to prevent collaterals from being developed.

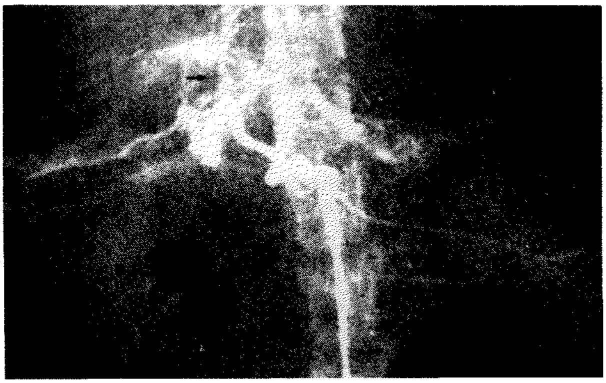

**Figure 4** Fully developed collaterals (arrow) 5 days after permanent dearterialization.

Ideally, dearterialization should only deliver ischemia to the tumours while leaving healthy liver free from damage. After permanent dearterialization or hepatic artery ligation liver enzymes are regularly released within 24 hours. The extent of necrosis depends on the degree and period of ischemia[15]. After transient dearterialization enzyme values are lower and come earlier depending on the duration of the arterial blockade[16]. Postponing the dearterialization till after the operation reduces the extent of liver damage as reflected by a lower release of enzymes. It is therefore not surprising that liver enzymes in our experiment remained within normal limits, thus, leaving the normal hepatic tissue without damage.

It seems, therefore, rationale to suggest that dearterializations for less than 3 hours will prevent the formation of collaterals. Moreover, separating the dearterializations from the initial operation should deliver selective ischemia to the liver tumours sparing the healthy liver tissue, and should also decrease morbidity and mortality otherwise associated with hepatic artery ligation or dearterialization.

### *References*

1. Mori, M., Masuda, M. and Miyanaga, T. (1966) Hepatic artery ligation and tumour necrosis in the liver. *Surgery*, **59**, 359–363
2. Nilsson, L.A.V. (1966) Therapeutic hepatic artery ligation in patients with secondary liver tumors. *Rev. Surg.*, **23**, 374–376
3. Nagasue, N., Murakami, H., Araki, S. *et al.* (1972) Hepatic artery ligation for metastatic leiomyosarcoma of the liver. *Jpn. J. Surg.*, **2**, 77–85
4. Bengmark, S. and Rosengren, K. (1970) Angiographic study of the collateral circulation to the liver after ligation of the hepatic artery in man. *Am. J. Surg.*, **119**, 620–624
5. Koehler, R.E., Korobkin, M. and Lewis, F. (1975) Arteriographic demonstration of collateral arterial supply to the liver after hepatic artery ligation. *Radiology*, **117**, 49–54
6. Persson, B.G., Jeppsson, B., Andersson, L. *et al.* (1987) The prevention of arterial collaterals after repeated temporary blockade of the hepatic artery in pigs. *World J. Surg.*, **11**, 672–677
7. Persson, B.G., Nobin, A., Ahrèn, B. *et al.* (1989) Repeated hepatic ischemia as a treatment for carcinoid liver metastases. *World J. Surg.*, **13**, 307–312
8. Persson, B.G., Jeppsson, B., Ekberg, H. *et al.* (1990) Repeated dearterialization of hepatic tumours with an implantable occluder. *Cancer*, **66**, 1139–1146
9. Dahl, E.P., Fredlund, P.E., Tylèn, U. *et al.* (1981) Transient hepatic dearterialization followed by regional intra-arterial 5-Fluorouracil infusion as treatment for liver tumours. *Ann. Surg.*, **193**, 82–88
10. Mack, P., Jeppsson, B., Rauszys, P. *et al.* (1989) Retarding liver cancer growth in the rat by transient repeated hepatic dearterialization. *J. Surg. Res.*, **46**, 123–128
11. El-Domeiri, A.A. and Mojab, K. (1978) Intermittent occlusion of the hepatic artery and infusion chemotherapy for carcinoma of the liver. *Am. J. Surg.*, **135**, 771–775
12. Wopfner, F. (1983) Intra-Arterial chemotherapy of the liver with transient, repeated hypoxia. *Cancer Res.*, **86**, 75–82
13. Michels, N.A. (1953) Collateral arterial pathways to the liver after ligation of the hepatic artery and removal of the celiac axis. *Cancer*, **6**, 708–724
14. McDermott, W.V. Jr., Paris, A.L., Clouse, M.E. *et al.* (1978) Dearterialization of the liver for metastatic cancer. Clinical, angiographic and pathologic observations. *Ann. Surg.*, **187**, 38--46
15. Frederiks, W.M., Bosch, J.J., Schröder, M.J.R., *et al.* (1982) A model for provoking ischemic necrosis in rat liver parenchyma and its quantitative analysis. *Exp. Pathol.*, **22**, 245–252
16. Jeppsson, B., Dahl, E.P., Fredlund, P.E. *et al.* (1979) Hepatic necrosis in the pig produced by transient arterial occlusion. *Eur. Surg. Res.*, **11**, 243–253

*(Accepted by L. Blumgart 6 March 1992)*

### INVITED COMMENTARY

Hepatic artery occlusion as a means of treatment for liver tumours has been pioneered in Lund. The experiment reported in this paper addresses the important question of how long the hepatic artery may be occluded, it has been found that up to four hours daily for five days does not induce collateral formation, as seen on coeliac angiography. The authors used a minaturised version of the Lund occluding cuff and have to be congratulated on overcoming the technical difficulties of constructing such a small cuff.

Presumably this work was done to provide background information for designing clinical studies using the cuff in tumour bearing patients. Seeking longer and longer ischaemic periods pre-supposes that it is the ischaemia which damages the tumour preferentially. It might well be, however, that re-perfusion is the important component and it would be interesting to see the effect of repeated short periods of occlusion, say 10 minutes occluded and 10 minutes un-occluded, repeated over a period of several hours.

No change in liver function tests were observed during the ischaemic episodes. It is also interesting that no evidence of hepatic artery thrombosis was seen, even with prolonged occlusion, except where infection occurred. There are two routes by which arterial collaterals could reach the liver during and after the occlusion, one is through existing channels (these were divided in this work), in the hepatic attachments and the authors mentioned the 26 channels reported by Michaels[1]. The other route is through new attachments or adhesions developing after surgery. Colaterals through the first route will require only a dilatation of existing vessels whereas the second route requires adhesions and the laying down of a neovasculature. Tumours are well known to be able to induce the formation of new blood vessels in their surroundings and I would very much like to see the series of experiments reported here repeated in a tumour bearing model, but this should be a liver metastasis model based on natural spread from a carcinogen induced colonic tumour or portal injection of tumour cells. A sub-capsular tumour implantation model might give less useful information in trying to understand the clinical situation as its blood supply might not correspond to clinical tumours.

### *Reference*

Michaels, N.A. (1953) Collateral arterial pathways to the liver after ligation of the hepatic artery and removal of the celiac axis. *Cancer*, **6**, 708–724

Malcolm Puntis
Department of Surgery
University of Wales College of Medicine
University Hospital of Wales
Cardiff CF4 4XN

